# Supplementary material for: Seroprevalence and preventive practices of dengue and chikungunya among school children in Bangkok: Gaps in prevention and vaccination strategies
Source: PLoS Negl Trop Dis. 2026 Mar 16;20(3):e0013026. doi: 10.1371/journal.pntd.0013026 (PMC13004517; doi:10.1371/journal.pntd.0013026)
Supplement: S1 Text — (PDF) [file pntd.0013026.s001.pdf]

The association between geographic area and mosquito-borne disease exposure risk in Bangkok

N°: 2023-136

Version 5.0 – 4 April 2024

Confidential

Laboratory	Ecology and Emergence of Arthropod-borne Pathogens<br>Institut Pasteur<br>25-28 rue du Docteur Roux<br>75724 Paris Cedex 15
Project leader	Richard PAUL<br>Institut Pasteur<br>Phone : 33(0)140613626<br>Fax : 33 (0)1 45 68 89 29<br>Email : rpaul@pasteur.fr
Local project leader	Watsamon Jantarabenjakul, MD, PhD.<br>Department of Pediatrics, Faculty of Medicine,<br>Chulalongkorn University<br>1873 Rama 4 Road, Pathumwan,<br>Bangkok, 10330 Thailand<br>Phone: +6622564930<br>Fax: +6622564930<br>Email: Watsamon.j@chula.ac.th
Co-Investigator	Thanyawee Puthanakit, MD<br>Department of Pediatrics, Faculty of Medicine,<br>Chulalongkorn University<br>1873 Rama 4 Road, Pathumwan,

	Bangkok 10330 Thailand<br>Phone: +6622564930<br>Fax: +6622564930<br>Email: thanyawee.p@chula.ac.th
Co-investigator<br><br><br><br><br><br>Co-Investigator<br><br><br><br><br><br><br>Co-Investigator<br><br><br><br><br><br><br><br>Co-Investigator	Padet Siriyasatien<br>Department of Parasitology, Faculty of Medicine, Chulalongkorn University,<br>1873 Rama 4 Rd, Pathumwan, Bangkok, 10330, Thailand<br>Phone : +6622564387<br>Email: padet.s@chula.ac.th<br><br>Ekasit Kowitdamrong, MD.<br>Department of Microbiology, Faculty of Medicine, Chulalongkorn University<br>1873 Rama 4 Road, Pathumwan,<br>Bangkok 10330 Thailand<br>Phone: +6622564132, Fax: +6622525952<br>Email: ekasit.k@chula.ac.th<br><br>Thitiya Yakasaem, MD<br>Pediatrics, Faculty of Medicine,<br>Chulalongkorn University<br>1873 Rama 4 Road, Pathumwan,<br>Bangkok, 10330 Thailand<br>Phone: +6622564930 Fax: +6652564930<br>Email: thitiya.y@chula.ac.th<br><br>Nattapong Jitrungruengnij, MD<br>Charoen Krung Pracharak Hospital<br>8 Charoen Krung Rd., Bang Kho Laem, Bangkok 10120, Thailand<br>and<br>Center of Excellence for Pediatric Infectious Diseases and Vaccines<br>Faculty of Medicine, Chulalongkorn University<br>1873 Rama 4 Rd, Pathumwan, Bangkok, 10330, Thailand<br>Phone: +6622564930, +66869850091<br>Email: Jitrungruengnij.n@gmail.com<br>Nattapong.ji@chula.ac.th

Study Coordinator	Thidarat Jupimai<br>Center of Excellence for Pediatric Infectious Diseases and Vaccines<br>Faculty of Medicine, Chulalongkorn University<br>1873 Rama 4 Rd, Pathumwan, Bangkok, 10330, Thailand<br>Phone : +6622564000 Ext.3362<br>Email : thidarat.j@chula.ac.th
Laboratory (Thailand)<br><br>Laboratory (Thailand)	Department of Microbiology, Faculty of Medicine,<br>Chulalongkorn University<br>15th -17th Floor, Aor Por Ror Building<br>1873 Rama 4 Road, Pathumwan<br>Bangkok, 10330 Thailand<br><br>Department of Parasitology, Faculty of Medicine,<br>Chulalongkorn University<br>18th Floor, Aor Por Ror Building<br>1873 Rama 4 Road, Pathumwan<br>Bangkok, 10330 Thailand
Head of Laboratory	
	Anavaj SAKUNTABHAI<br>adresse si différente IP<br>Phone: 33 (0)1 44 38 91 03 Fax : 33 (0)1 45 68 89 29<br>Email : anavaj@pasteur.fr

Other partners(s)

Geo-localisation

Eric Daudé & Alexandre Cebeillac
Institut de recherche sur l'Asie du Sud-Est contemporaine IRASEC, CNRS
179 Thanon Witthayu, Lumphini
Pathumwan, Bangkok 10330 Thailand

SUMMARY of PROTOCOL

1. Title	The association between geographical area and mosquito-borne diseases exposure risk in Bangkok
2. Acronym	GeoMosquito
3. Type of study	A cross-sectional school-based seroprevalence study among children aged 10-15 years. Sero-conversion to dengue virus and chikungunya virus exposure will be measured by the Dengue IgG Rapid Diagnostic kit and the Chikungunya IgG Rapid Diagnostic kit, respectively.<br>A cross-sectional questionnaire study. Questionnaire about KAP about mosquito-borne diseases among their parents who enrolled in the seroprevalence study<br>A prospective observational study of mosquito sampling study in schools and in the homes of 10% of the recruited children using sticky traps
4. Rationale	Specific places have the potential for being hotspots of viral transmission because of their conduciveness for mosquitoes and their environmental and demographic characteristics that enable these mosquitoes to find and bite their hosts. These areas are predicted to be responsible for the multiplication of cases at a hyperlocal level. Places that are hyper-connected (by transport networks) contribute significantly to viral spread at a city-wide scale, not least from the above-mentioned hotspots. We hypothesize that hyper-connected intra-urban districts will have a higher exposure to dengue and chikungunya, dependent upon the predicted environmental suitability for mosquitoes.
5. Principle objective	Assess the extent of dengue virus and chikungunya virus seropositivity, using Rapid Diagnostic tests for IgG in ~1000 school children, aged 10-15 years of age, spread across 2-3 schools of four different environments classified by their degree of connectivity and extent of built-up area, is associated with highly connected areas and predicted high mosquito abundance areas within Bangkok.
6. Secondary objectives	Collect mosquito samples in these 8-12 schools<br>Assess knowledge, attitude, and practice about mosquito-borne disease, control measures, and vaccine acceptance among parents
7. Principle criteria of evaluation	Seroprevalence rates
8. Secondary criteria	Mosquito numbers<br>Answers to a questionnaire about knowledge, attitude, and practice

	about mosquito-borne disease, control measures, and vaccine acceptance among parents
9. Subjects and numbers of subjects	~ 1000 children 10-15 years of age for DENV and CHIKV seroprevalence study<br>~ 1000 parents of children from the seroprevalence study for a questionnaire study<br>~ 100 homes for mosquito sampling study
10. Inclusion criteria	- Acceptance to participate
11. Exclusion criteria	- Refusal to participate or suffering from any concurrent infection or with any physical or mental disability.
12. Material/ Biological samples	- Finger-prick blood sample for use with Rapid Diagnostic Kit<br>- Adult mosquitoes
13. Individual data collection and flow	For each participating child:<br>- information on age, sex and history of infection<br>- place of residence (longitude & latitude)<br>KAP questionnaire for parents/care givers – age, sex, address, education level
14. Study timeline	Estimated start date of enrollment: 01/06/2024<br>Enrollment period: 6 months<br>Duration of subject's participation:<br>- 4 months for mosquito sampling study<br>- 1 day for seroprevalence study and questionnaire<br><br>Total estimated study duration: 1 year<br>Data analysis: 6 months<br>Data storage period: 2 years after the last publication
15. Number of centres	8-12 schools
16. Expected results	Sero-prevalence rates for dengue and chikungunya stratified by age, sex and environmental typology (4 types: High vs. Low centrality and High vs Low vegetation) of home and school. Mosquito abundance according to environmental typology. KAP on dengue and chikungunya.

PAGE DE SIGNATURES

I have read the protocol carefully and I consider that it contains all information necessary to undertake the study. I will carry out the study according to the protocol and abide by the appropriate legislative rules.

Head of Laboratory

Anavaj Sakuntabhai, Head of Unit

Date : 28 August 2023

Signature :

Project leader

Richard Paul, Senior Staff Scientist

Date : 28 August 2023

Signature :

Clinician

Watsamon Jantarabenjakul, Co-Principle Investigator

Date : 4 Sep 2023

Signature :

Watsamon Jantarabenjakul

SUMMARY

SUMMARY OF PROTOCOL 4

PAGE DE SIGNATURES 6

SUMMARY 7

1. SCIENTIFIC JUSTIFICATION OF THE RESEARCH 9

Background 9

2. OBJECTIVES 12

3. METHODOLOGY 12

3.1. Type of research 12

3.2. Evaluation criteria 12

4. STUDY POPULATION AND SOURCE OF RECRUITMENT 12

4.1. Inclusion criteria 13

4.2. Exclusion criteria 13

4.3. Source of recruitment 13

5. PRACTICALITIES OF THE RESEARCH STUDY 14

5.1. General organisation of the study 14

5.2. Compensation 14

5.3. Benefits to the individual and the community 15

6. BIOLOGICAL SAMPLE COLLECTION 15

6.1. Samples description 15

6.2. Sample flow 15

6.3. Biological analyses 15

6.4. Human genetic analyses (if applicable) 15

6.5. Secondary use of samples 15

7. DATA COLLECTION AND DATA MANAGEMENT 16

7.1. Data collection 16

7.2. Data management 16

8. STUDY CALENDAR 17

9. STATISTICAL CONSIDERATIONS 18

10. REGLEMENTARY ASPECTS OF THE STUDY 18

10.1. Methods for information and consent for study participants 19

10.2. Data confidentiality 19

10.3. Vigilance 19

10.4. Insurance 20

11. STEERING COMMITTEE 20

12. DISSEMINATION OF THE RESULTS 20

12.1. Dissemination of the results to the participants 20

12.2. Rules for Publishing 20

13. ARCHIVING 21

14. BUDGET 21

15. BIBLIOGRAPHY 21

1. SCIENTIFIC JUSTIFICATION OF THE RESEARCH

Background

Mosquito-borne diseases are those spread by the bite of an infected mosquito. *Aedes* spp. can transmit dengue virus (DENV), chikungunya virus (CHIKV), zika virus and yellow fever virus. In Thailand, dengue and chikungunya pose a significant public health concern, particularly among adolescents. Dengue fever is a disease that has no specific antiviral treatment but is managed supportively. Currently, the care and treatment of dengue fever patients has become much more effective. However, it is still observed that the mortality rate is approximately 0.1 to 0.5 percent, resulting in significant losses. Chikungunya is a viral illness characterized by sudden onset of high fever, joint pain, rash and myalgia. It can cause persistent joint pain that may last for months or years among adults but mild joint pain in children. However, neurological complication is reported frequently among children. There is also no specific antiviral treatment for chikungunya, so management focuses on supportive care to alleviate symptoms.

Prevention of dengue and chikungunya infection involves measures to reduce mosquito breeding/oviposition sites, and personal protective measures, including the use of mosquito repellents and wearing long-sleeved clothing, are also recommended. Currently, dengue vaccines are available and can prevent infection and decrease severity, but remain controversial because of potential increased severity upon infection with DENV in naïve individuals – the phenomenon known as Antibody-dependent enhancement. There is no available chikungunya vaccine.

Dengue and chikungunya are associated with a wide range of socio-economic factors that alter the risk of exposure to infectious mosquitoes and which can vary at very local scales [1-8]. However, these associated risk factors are not systematic [9,10], likely influenced by the role of human mobility in ferrying the virus from places of high environmental risk throughout the city [11-13]. Indeed, low risk mosquito abundance areas do not necessarily lead to reduced exposure to virus and the key factor seems to be the extent of connectedness of an area. Analyses conducted in Bangkok have identified specific socio-economic factors associated with dengue cases, but whose association with dengue incidence radically alters and improves when factoring in the Bangkok transport matrix (connectivity among the 180 Khwaeng) and differs from when just using physical distance (Table 1). However, clinical dengue cases make up only a fraction of the true number of DENV infections and may be subject to reporting bias. A more robust measure of exposure is therefore using serology surveys across a wide range of age groups.

Table 1. Effect of matrices of distance and transport connectivity among subdistricts on association of SE variables with dengue cases in Bangkok. Shown are parameter estimates and percentage of variation explained in the multivariate statistical analyses.

	Transport matrix		Distance matrix	
	w/o	with	w/o	with
No education	0.047	0.081	0.047	0.072
	2.2%	36.9%	2.1%	73.1%
% Cement houses	0.0035	0.0045	0.0034	-0.027
	11.5%	36.9%	10.8%	0.03%
N° houses (100s)	0.0023	0.0023	0.002	0.0036
	46.6%	18.9%	48.1%	23.3%

As a pilot study, prior to roll-out across Bangkok, dengue virus sero-prevalence rates were measured using the Panbio Indirect IgG ELISA on stored blood samples from 272 children aged 3-15 years from within Bangkok. These were mapped according to their homes and schools, whose environmental typology (satellite imagery for vegetation index, height and surface of buildings) and degree of centrality (services density, frequency of transportation and visits using social media data) were established (see Figures below). The association of the degree of centrality (high *vs*. low) and the type of environment (Open *vs*. densely built-up) with sero-prevalence rates was then analysed by logistic regression, taking into account the ages of the individuals.

From this retrospective study, the median (IQR) age of the samples was 10.7 (7.6-12.6) years. DENV sero-prevalence rates among children 3-9 years and 10-15 years were 6.5% and 37.2% respectively. Children going to schools characterized by an open environment or one of low centrality had much lower sero-prevalence rates (Open *vs*. dense: 16.8% *vs*. 27.1%; Low vs high centrality: 14.8% vs. 25.2%). There was no difference in sero-prevalence rates according to the degree of centrality of their homes. However, sero-prevalence rates in homes in open environments were higher (27.1% *vs*. 12.6%) (Fig. 3).

Figure 3. Sero-prevalence rates according to landscape category and centrality in children's homes and schools

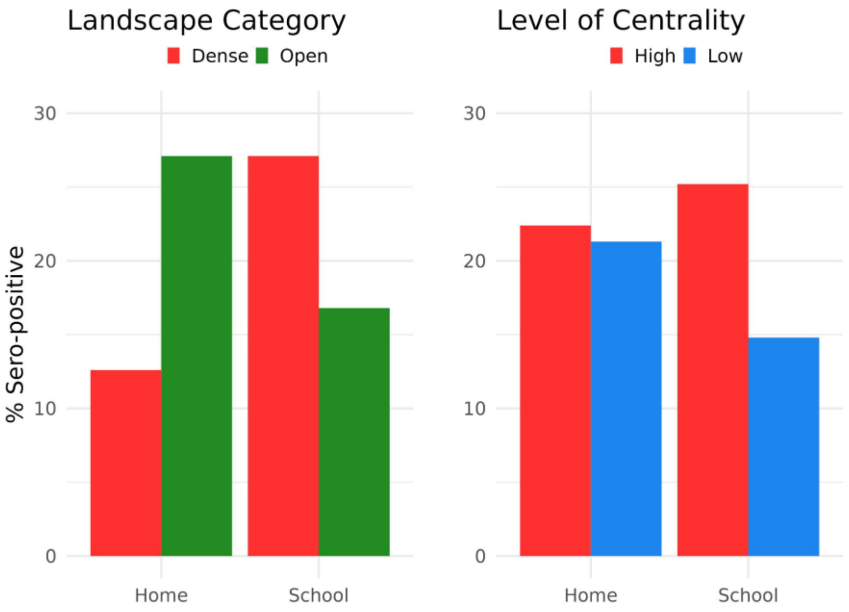

Suwanbamrung et al. [14] conducted a cross-sectional study among children aged 8-13 years from 50 public schools in Kanchanadit district to assess the correlation between knowledge, attitude and practice (KAP) in dengue infection including prevention method compare between children with and without dengue infection experience using 32-items questionnaire. The study shows no significant difference in KAP mean score between the 2 groups, but the children with previous dengue infection had better preventive practice than the other group (OR 1.34, 95%CI 1.03-1.75, P=0.032). The study also notes that most children (more than 70%) reported that their source of dengue information came from their teacher. Thus, in addition to the sero-prevalence study, we are also interested in assessing levels of KAP concerning dengue and provide more information about dengue infection and prevention, which can then be applied in their school and household.

Vongpunsawad et al. [15] conducted sero-prevalence surveys of CHIKV and DENV in Thailand, in which 835 serum samples were obtained from individuals living in central and southern Thailand by using anti-CHIKV and anti-DENV IgG antibodies commercial enzyme-linked immunosorbent assays. Overall seropositivity of CHIKV in children aged < 15 years was 10.3%, of which 17.8% among participants from the south and 2.6% among participants from central Thailand. Seropositivity of DENV was 31.9% among children aged < 9 years in both in the southern and central parts, 55.4% among children aged 10-19 years among southern participants and 62.5% among children aged 10-19 among central participants. The current situation in Bangkok is unknown, but a large scale outbreak occurred all of Thailand in 2018-2019 and *Aedes aegypti* was incriminated as the major vector [16]. We thus anticipate that the sero-prevalence rates will be influenced by the environmental typology as for DENV.

Based on these preliminary findings, this current project aims to expand this sero-prevalence study in a prospective school-based study in children aged 10-15 years old for both DENV and CHIKV infections.

2. OBJECTIVES

Primary objective:

To address the extent to which (satellite-) predicted and empirically validated mosquito abundance and mapped transport/mobility connectedness among Khwaeng do associate with DENV and CHIKV exposure as measured by sero-positivity. Coupled with a detailed geographic information system (GIS) of Bangkok, including fine scale meteorological data, will subsequently enable fine scale agent-based simulation models to test *in silico* the efficacy of implementing vector control in a variety of locations.

Secondary objective:

To assess knowledge, attitude, and practice (KAP) about mosquito-borne disease, control measures, and vaccine acceptance among parents

3. METHODOLOGY

3.1. Type of research

This study divides into 3 parts

1. A cross-sectional school-based sero-prevalence study among children aged 10-15 years. Sero-conversion to dengue virus and chikungunya exposure will be measured by Dengue IgG Rapid Diagnostic kit and Chikungunya IgM/IgG Rapid Diagnostic kit, respectively (SD Bioline).
2. A cross-sectional questionnaire study. The questionnaire about KAP about mosquito-borne diseases among their parents who enrolled in sero-prevalence study
3. A prospective observational study of mosquito sampling study in schools and in the homes of 10% of the recruited children using sticky traps

3.2. Evaluation criteria

Principle evaluation criteria:

Epidemiological read-out – sero-prevalence

To assess the association of centrality with exposure to DENV and CHIKV, we will perform a sero-prevalence study using Rapid Diagnostic kits on children 10-15 years spread across 8-12 schools in Bangkok.

Secondary evaluation criteria:

Entomological read-out. Adult mosquito numbers caught on Gravid Aedes Traps (sticky paper) weekly throughout the year in the schools and during the summer months in a sample of children's homes.

Answers to a questionnaire about knowledge, attitude, and practice about mosquito-borne disease and control measures among parents

4. STUDY POPULATION AND SOURCE OF RECRUITMENT

4.1. Inclusion criteria

1. Aged 10-15 years old from schools characterised by the environmental and centrality typology.
2. Signed informed consent.

4.2. Exclusion criteria

1. Suffering from any concurrent infection or with any physical or mental disability.
2. Refusal to participate.

4.3. Source of recruitment

Children of ages 10-15 years old from 8-12 schools will be invited to participate. The schools will be chosen from a list (below) based on their environmental and centrality typology.

id_sch	name	type	nb_stu	class
3110023402	โรงเรียนรัตนจีนะอุทิศ	BMA	420	central-_env-
3110015008	โรงเรียนชูสินทองประดิษฐ9อนุสรณ9	BMA	152	central-_env-
10032003	โรงเรียนสุวรรณพลับพลาพิทยาคม	OBEC	671	central-_env-
3110010609	โรงเรียนวัดธรรมาภิรตาราม	BMA	133	central-_env-
3110012104	โรงเรียนวัดนวลจันทร9	BMA	205	central-_env-
3110013706	โรงเรียนคลองกลันตัน	BMA	173	central-_env-
10032029	โรงเรียนแจงรFอนวิทยา	OBEC	411	central-_env-
10032012	โรงเรียนมหรรณพาราม	OBEC	1200	central-_env-
3110014705	โรงเรียนอำนวยกนกศิริอนุสรณ9	BMA	200	central-_env+
3110013001	โรงเรียนวัดราชสิทธาราม	BMA	110	central-_env+
10032013	โรงเรียนชิโนรสวิทยาลัย	OBEC	2079	central-_env+
10032030	โรงเรียนวัดบวรมงคล	OBEC	400	central-_env+
3110022710	โรงเรียนวัดมะลิ	BMA	176	central-_env+
3110012003	โรงเรียนเทพวิทยา	BMA	217	central-_env+
3110024503	โรงเรียนสุเหราบFานดอน	BMA	106	central-_env+
3110012914	โรงเรียนวัดรัชฎาธิฐาน	BMA	135	central-_env+
3110020303	โรงเรียนวัดปทุมวนาราม ในพระบรมรา ชูปถัมภ9สมเด็จพระเทพรัตนราชสุดาฯ สยามบรมราชกุมาริ	BMA	291	central+_env-
10010003	โรงเรียนพระตำหนักสวนกุหลาบ	OBEC	287	central+_env-
3110011202	โรงเรียนวัดราชสิงขร	BMA	444	central+_env-
10012012	โรงเรียนเบญจมราชาลัย ในพระราชอุปถัมภ9	OBEC	1881	central+_env-
3110011304	โรงเรียนวัดธรรมมงคล (หลวงพIอวิริยังค9อุปถัมปR)	BMA	140	central+_env-
3110011102	โรงเรียนวัดยานนาวา	BMA	110	central+_env-
10012030	โรงเรียนวัดสุทธิวราราม	OBEC	2899	central+_env-
3110011904	โรงเรียนวัดศรีบุญเรือง	BMA	180	central+_env-
3110014508	โรงเรียนสวัสดีวิทยา	BMA	178	central+_env+
10012009	โรงเรียนวัดบวรนิเวศ	OBEC	245	central+_env+
10012001	โรงเรียนสตรีวิทยา	OBEC	2917	central+_env+
3110011002	โรงเรียนวัดชIองลม	BMA	192	central+_env+
10012015	โรงเรียนวัดสังเวช	OBEC	170	central+_env+
3110011903	โรงเรียนวัดเทพลีลา	BMA	392	central+_env+
10022005	โรงเรียนเทพลีลา	OBEC	2115	central+_env+
10012025	โรงเรียน ไตรมิตรวิทยาลัย	OBEC	919	central+_env+
10012010	โรงเรียนวัดราชบพิธ	OBEC	1789	central+_env+
3110010904	โรงเรียนวัดทัศนารุณสุนทริการาม	BMA	107	central+_env+

5. PRACTICALITIES OF THE RESEARCH STUDY

The main objectives of this study are to study the sero-prevalence of previous dengue and Chikungunya infection in 4 types of areas categorised by their centrality and degree of vegetation using rapid diagnostic IgG test kits. A second aim is to measure the mosquito abundance in schools and homes categorised by their area type. A final aim is to assess knowledge, attitudes, and practices regarding preventing Dengue fever and Chikungunya.

5.1. General organisation of the study

A. Sero-prevalence

A one-time cross-sectional sero-prevalence study to measure IgG seropositivity will be performed in each of the selected schools in pupils aged 10-15 years old. The total number of participants in the research project is 1000. Collected from 2-3 schools in each of the four area types.

After consent to allow child participation, the pediatrician will use a rapid diagnostic lateral flow blood test to measure the immunity against dengue and chikungunya from the children. The blood sampling will be drawn from the fingertips for two drops (1 drop per test) at the child school.The result will be reported within the same day of the test. Parents will be required to answer the Dengue and Chikungunya questionnaires.

B. Mosquito collections

BioGents Gravid Aedes Traps that capture adult *Aedes aegypti* via a sticky insert, will be deployed in each of the schools and monitored weekly for one year.
In addition, based on the four area types, 100 houses will be randomly selected to collect samples of *Aedes aegypti* mosquitos at home during the rainy summer period May to September. 100 children will be provided with a mosquito-trapping device. For trapping mosquitoes, we will use Gravid Aedes Traps (GATs) with a sticky insert to catch and count mosquitoes. GATs are essentially a black plastic bucket (with a hole two-thirds way up to let excess rainwater flow out) in which we place water with hay/dried grass. A double netting prevents the mosquitoes from actually laying their eggs in the hay infusion and a sticky insert glues the mosquitoes. These traps will be placed in areas protected from rain and direct sunlight. Depending on whether the school is open to the outside or not, GATs will be placed indoors and outdoors. Likewise for the homes, GATs will be placed such that they will not get knocked over/stolen. GATs will be monitored daily by school teachers/students to ensure they do not dry out, are attacked by ants/geckos etc and the sticky strip will be replaced once a week and placed into a ziplock type plastic bag with information on date and address written in indelible ink on the bag. The bags will then be stored in a reasonably cool room and then collected by the entomologists within a reasonable delay.

C. Questionnaire

A questionnaire about knowledge, attitude and practice about dengue infection, chikungunya infection, prevention, and control measure will be administered to the parents/care givers of the participating children (1000).

5.2. Compensation

Parents will receive 500 Baht for answering the KAP survey.

Children (100) participating in the mosquito collections at their homes will receive 1000 Baht per month for a maximum of 4 months.

5.3. Benefits to the individual and the community.

The major benefit of the study is to generate first insight into the relationship between place of school and residence (defined by centrality and environmental typology) and risk of exposure to DENV and CHIKV. The study will also generate sero-prevalence data, which can then guide the local public health authorities for any potential implementation of the Takeda QDENGA®▽ (Dengue Tetravalent Vaccine [Live, Attenuated]) under evaluation for authorisation in Thailand. Knowledge of sero-positivity to the dengue virus will also be of direct benefit to the individual, especially with regard to the above-mentioned vaccine. Moreover, data from KAP about mosquito-borne diseases, control measures, and vaccine acceptance can contribute to the public health prevention campaigns concerning mosquito-borne diseases.

6. BIOLOGICAL SAMPLE COLLECTION

6.1. Samples description

A. biological samples of human

Finger-prick blood samples for use on a Rapid diagnostic lateral flow test (RDT) from 1000 children. No samples will be kept.

B. biological samples of mosquitoes

Mosquitoes caught on the sticky traps in the schools and homes will be identified to gender and species. There is no use of insecticides in these traps, just glue on paper.

6.2. Sample flow

A. Labelling

The mosquito sticky paper catches will be changed weekly and placed into a ziplock bag with the date and address written in indelible ink on the outside of the bag and collected as soon as possible (days) by the entomological team.

B. Shipment

The mosquito sticky catches will be transferred to the entomology laboratory at Chulalongkorn University and identified to species and gender. Once identified and entered into the computer the samples will be disposed of. Once the mosquito sampling is complete, as above, the school/house address will be transformed into one of the four environmental categories. The address will then be erased.

C. Storage

There will be no storage of samples.

6.3. Biological analyses

All RDT analyses will be performed on site in the schools. Mosquitoes will be identified by the entomologists of Chulalongkorn University. The RDTs and the mosquito samples will be disposed of once the results are known.

6.4. Human genetic analyses (if applicable)

None

6.5. Secondary use of samples

None

7. DATA COLLECTION AND DATA MANAGEMENT

7.1. Data collection

For connectivity and environmental typology of the area within which the individual goes to school and lives

As shown above (Figs 1 & 2), Bangkok has previously been mapped using Twitter, Google POI, Facebook and transport information to define the centrality level of each sub-area and satellite images and built-up databases for its environmental typology.

For sero-conversion study (specific data collection):

For each participating child, the age, gender, history of infection/ dengue vaccination, school name and address, house address and RDT test results for both dengue and Chikungunya will be written on the Case Report Form (CRF) immediately following the test. information on age, sex and history of infection/ dengue vaccination will be obtained. We will also collect the school and place of residence coordinates (longitude & latitude), which will be subsequently classified in one of the four typologies Central+/Environmental+, Central+/Environmental-, Central -/Environment+, Central-/Environment aggregated to the scale of the grids as shown in Figures 1 & 2 and the coordinates information then erased.

For mosquito data

BioGents Gravid Aedes Traps will be implemented in all participating schools for a period of one year and monitored weekly. These traps use sticky paper (glue) to capture flying adult mosquitoes. There is no use of insecticides. From May to September children in these schools will also be invited to take a trap home and collect mosquitoes in / around their homes.

For weather data

We will use i-Buttons affixed to the GAT traps to measure temperature and relative humidity.

For questionnaire data

Answers about KAP about mosquito-borne disease as Yes/No or composited score which will be standardized to a scale of 0-100%; 80-100% is considered a high score, 50-80% a moderate score and 50% or below a low score.

7.2. Data management

The data will be input twice into two separate Xcel files and cross-checked. The address will be geolocated and then transformed to one of the four environmental categories before being erased.

For all types of data (sero-prelance, mosquito and KAP), once the data has been entered (double entry and cross-check), all paper forms will be destroyed.

The sero-prevalence data will be maintained and analysed in the Pediatric department, Chulalongkorn Universty. The list of school and home addresses (coordinates) will be transformed into environmental typology by the IRASEC team present in Bangkok and this typological information sent back to the Pediatric department and linked to their sero-prevalence database. Once this link has been made and double checked, the coordinates information will be erased. The Institut Pasteur team will perform the statistical analysis with the Pediatric team in Chulalongkorn as described below.

The mosquito data will remain in the entomology department, Chulalongkorn University, and as above the coordinates linked to the environmental typology and subsequently analysed as described below.

The KAP data will be maintained and analysed in the Pediatric department, Chulalongkorn Universty. Once entered into the computer the address will be erased and the analysis perfomed as described below.

Only aggregated (analysed) data will be transferred to France for publication writing.

Limited data from the professionals in charge of the clinical investigation and its organization	
**Data**	**Justification**
Personal data: last name, first name, e-mail, telephone number, postal address, guardian/trusted person...	Contacting participants to set up appointments, returning overall and/or individual results, maintaining communication with participants during the research, exercising participants' rights.

Data available to investigators and scientists for research	
**Data**	**Justification**
**Identification data :** participant code	Linking a participant's data set and samples
**Demographic data:** age, gender, level of instruction,	Describe the study population
**Medical data:** history of dengue and chkungunya infection, dengue vaccination and RDT test results for both dengue and chikungunya	Describe the general clinical pattern of the subjects studied for sero-conversion study.
**Localisation:** school name and address, home address, environmental typology	Connectivity and environmental typology of the area within which the individual goes to school and lives
**Assessement** on dengue and chikungunya among parents	Describe the knowledge, attitude, and practice about mosquito-borne disease, control measures, and vaccine acceptance

8. STUDY CALENDAR

Estimated start date of enrollment: 01/06/2024

Enrollment period: 6 months

Duration of subject's participation:

- 4 months for mosquito sampling study

- 1 day for seroprevalence study and questionnaire

Total estimated study duration: 1 year

Data analysis: 6 months

Data storage period: 2 years after the last publication before archiving the data base. Data will be kept at the Chulalongkorn University, 2 years after the last publication of the results estimated at 4 years after enrolment initiation and then archived. No individual data will be stored at Institut Pasteur.

9. STATISTICAL CONSIDERATIONS

Sample size:

Based on the preliminary data showing a difference in prevalence rates according to school centrality and environmental typology (approximately 26% vs 15%), we estimate a necessary sample size of ~210 children for each of the four typologies (Central+/Environmental+, Central+/Environmental-, Central -/Environment+, Central-/Environment-).

Sample size was calculated as follows [17]

$n = (Z_{\alpha/2} + Z_{\beta})^2 . [p_0(1 – p_0) +p_1(1-p_1)] (p_0-p_1)^2$

Where n is the required sample size per treatment arm, Zα and Zβ are constants set by convention according to the accepted α error and whether a one-sided or two-sided effect, p0 is the proportion sero-positive in area type 0 (ie Central + or Environment +), and p1 the proportion areas type 2 (Central – or Environment -).

Assuming a $p < 0.05$ as acceptable and a study with 90% power, the following constant values are: $Z_{\alpha/2} = 1.96$ and $Z_{\beta} = 0.84$.

Because our preliminary data indicated variation within school centrality and environmental typology, (coefficient of variation ranging from 0.6-1), we used the following formula to estimate the number of different sites for each typology.

Site number per typology = $1+ (Z\alpha/2 + Z\beta)^2 . [p_0(1 – p_0)/n +p_1(1-p_1)/n +k^2(p_0^2+p_1^2)] (p_0-p_1)^2$

Where k is the coefficient of variation and n the number of individuals as calculated above. Using n=210 and k=0.6 to 1, we would need at least two and optimally three schools per environmental typology with 80-100 children per school.

Sero-positivity (as defined by the manufacturers instructions) will be analysed by logistic regression with age, sex and centrality (high vs. low) and environmental typology (Open vs Dense) as explanatory variables using R. School mosquito densities will also be included as an explanatory variable. In the subset of individuals collecting mosquitoes in the homes will be taken into account in a separate analysis. Mosquito densities will also be analysed with respect to the environmental typology and meteorological parameters.

Socio-demographic and other baseline characteristic variables of a questionnaire will be reported descriptively using counts and percentage for categorical variables and means for continuous variables. Comparison of willingness to be vaccinated and other associated factors will be performed using one-way analysis of variance. Multivariate regression analysis will be deployed to identify and understand the key factors that may be associated with the potential uptake a vaccine using generalized linear models.

10. REGLEMENTARY ASPECTS OF THE STUDY

Typology: Prospective analyses and Cross-sectional study

Regulatory Framework: Research must respect the Chulalongkorn University regulatory framework, international recommendations (Revised Declaration of Helsinki, 2013, CIOMS International Guidelines for Health-Related Human Behavioral Research, 2016) as well as the French regulations on data protection (Act N ° 78-17 relating to the protection of individuals with regard to the processing of personal data, 2016).

Regulatory procedures:

As a consequence of the regulatory framework mentioned above, it will be necessary to obtain:

- a favorable opinion from the Chulalongkorn University Committee of Ethics
- a favorable opinion from the IRB of the Institut Pasteur
- CNIL authorization in France if it does not comply with a reference methodology (conformity to be verified)
- there is no importing of human or mosquito biological samples or corresponding individual data into France.

10.1. Methods for information and consent for study participants

The project will be explained to the children and a notice of information in Thai about the purposes of the study given to them and their parents. Recruitment will only occur once the parents / care givers have signed the written informed consent form and the children an assent form in the presence of an independent witness (school teachers).

10.2. Data confidentiality

Once the RDT result and the grid level area of residence is established, the identification of the individual who provided the samples will no longer be possible. The identity of individual participants will be replaced with a code as follows:
Individual : XX-YYY-P/C

XX: School number

YYY: Individual number within the school

P/C: Parent or Child

Example: School 1, Individual 2, Child: 01-02-C

Correspondance between individual address and sample until geolocalisation enables environmental typology categorisation

10.3. Vigilance

Potential risks

Risks from taking a finger-prick blood sample: bleeding, bruising, swelling at the site of the finger-prick, fainting. If any side-effects occur, seek medical attention from our doctors who will provide appropriate treatment. If associated symptoms are the result of this research project, treatment will be free of charge.

Serious adverse event

Any untoward medical occurrence, unintended disease or injury or any untoward clinical signs (including an abnormal laboratory finding) in subjects, users or other persons whether or not related to the procedures involved (any procedure in the clinical investigation plan).

An adverse event/reaction must be considered as serious when it:

- results in death,
- is life-threatening,
- requires hospitalisation or prolongation of existing hospitalisation
- results in persistent or significant disability or incapacity,
- is a congenital anomaly or birth defect,
- is medically important (Some medical events may jeopardise the subject or may require an intervention to prevent one of the above characteristics/consequences.).

Regarding a life- threatening event, this refers to an event in which the subject was at risk of death at the time of the event; it does not refer to an event which hypothetically might have caused death if it were more severe

The following events will not be considered as serious:

- A planned hospitalization for pre-existing condition, or a study-specific procedure described in the protocol, without a serious deterioration in health
- Hospitalisation for a duration less than 24 h (i.e. not requiring a night in the hospital), unless it could be considered as an important medical event,
- Hospitalisations which have been planned before the subject's participation in the protocol,
- Hospitalisation for the treatment of a concomitant pathology diagnosed before the subject's participation in the protocol, except if the treatment intensity or frequency is increased during the subject's participation in the protocol,
- Hospitalisation for routine clinical procedures as well as on social grounds.

All serious adverse events experienced during the study, which are observed by the investigator or reported by the patient, will be recorded and declared to IRB.

10.4. Insurance

This project using a RDT is considered being at very low risk, but insurance is required.

11. STEERING COMMITTEE

Xavier Rodo : ISGlobal, Barcelona xavier.rodo@isglobal.org

Kanchana Nakhapakorn: Mahidol University, Thailand kanchana.nak@mahidol.ac.th

Joacim Rockloev: Heidelberg University joacim.rockloev@uni-heidelberg.de

Role: Independent consultants

12. DISSEMINATION OF THE RESULTS

12.1. Dissemination of the results to the participants

Information concerning the results of this study will be communicated during a session at each of the schools to which the participating children and parents will be invited.
Individual communication to the patient of their results is planned as part of this study as the result from the rapid diagnostic test is instantaneous (a matter of minutes) and the child will still be present when the result is known.

12.2. Rules for Publishing

The results of the study may be published in scientific journals after validation by a reading committee, or presented at scientific or medical meetings or congresses. The rules of authorship will follow the international rules of the ICMJE. These publication arrangements may be supplemented by contractual means between the different participants in this research. Concerning collaborations with the various partners of the study, the list of signatories and people thanked in the articles to be submitted will be established after collegial discussion. Institut Pasteur and more particularly the Clinical Research Coordination Center must be mentioned as responsible for ethical-regulatory and operational research activities.

13. ARCHIVING

The following documents will be archived for 15 years following the completion of the project:

- Protocol signed by the research partners (an original kept with each of the partners and an original of all the partners with the initiator of the research)
- Observation book / data collection medium (original at the initiator of the research and copied to the investigators and / or other collaborators)
- Final report of the study

14. BUDGET

This project is financed by a French National Funding Agency (Agence Nationale de la Recherche) grant awarded to R. Paul and E. Daudé for the Dengue RDT sero-survey and a Wellcome Trust grant to R. Paul for the mosquito collections [Total 2.2 million Thai Baht (56 000 euros)]. In addition, Chikungunya RDT sero-survey and Questionnaires study is supported by Ratchadapisek Sompoch Endowment Fund. [Total 400,000 Thai Baht]

15. BIBLIOGRAPHY

1. Misslin R, et al. Urban climate versus global climate change-what makes the difference for dengue?: Climate, dengue, and urban heat islands. Annals of the New York Academy of Sciences 2016; 1382(1): 56–72. https://doi.org/10.1111/nyas.13084
2. Telle O, et al. The Spread of Dengue in an Endemic Urban Milieu--The Case of Delhi, India. PLoS One 2016;11(1):e0146539. doi: 10.1371/journal.pone.0146539. PMID: 26808518.
3. Nagao Y, et al. Climatic and social risk factors for *Aedes* infestation in rural Thailand. Tropical Medicine International Health 2003; 8(7): 650–659. https://doi.org/10.1046/j.1365-3156.2003.01075.x
4. Tipayamongkholgul M, Lisakulruk S. Socio-geographical factors in vulnerability to dengue in Thai villages: a spatial regression analysis. Geospatial Health 2011; 5(2): 191–198. https://doi.org/10.4081/gh.2011.171
5. Kikuti M, et al. Spatial Distribution of Dengue in a Brazilian Urban Slum Setting: Role of Socioeconomic Gradient in Disease Risk. PLoS Negl Trop Dis 2015; 9(7): e0003937. https://doi.org/10.1371/journal.pntd.0003937
6. Zellweger RM, et al. Socioeconomic and environmental determinants of dengue transmission in an urban setting: An ecological study in Nouméa, New Caledonia. PLoS Negl Trop Dis 2017; 11(4): e0005471. https://doi.org/10.1371/journal.pntd.0005471
7. Farinelli EC, et al. Low socioeconomic condition and the risk of dengue fever: A direct relationship. Acta Tropica 2018; 180: 47–57. https://doi.org/10.1016/j.actatropica.2018.01.005
8. Jain R, et al. Prediction of dengue outbreaks based on disease surveillance, meteorological and socio-economic data. BMC Infect Dis. 2019; 19(1): 272. https://doi.org/10.1186/s12879-019-3874-x
9. Teixeira MG, et al. Exposure to the risk of dengue virus infection in an urban setting: ecological vs individual infection. Dengue Bulletin 2007; 31: 36–46.
10. Stewart-Ibarra AM, et al. Spatiotemporal clustering, climate periodicity, and social-ecological risk factors for dengue during an outbreak in Machala, Ecuador, in 2010. BMC Infect Dis. 2014 Nov 25;14:610. doi: 10.1186/s12879-014-0610-4. PMID: 29253873
11. Mondini A, Chiaravalloti-Neto F. Spatial correlation of incidence of dengue with socioeconomic, demographic and environmental variables in a Brazilian city. Science

of The Total Environment 2008; 393(2–3): 241–248. https://doi.org/10.1016/j.scitotenv.2008.01.010
12. Stoddard ST, et al. The role of human movement in the transmission of vector-borne pathogens. PLoS Negl Trop Dis 2009; 3(7):e481. doi: 10.1371/journal.pntd.0000481. PMID: 19621090
13. Stoddard ST, et al. House-to-house human movement drives dengue virus transmission. Proc Natl Acad Sci USA 2013; 110(3):994–999. doi: 10.1073/pnas.1213349110. PMID: 23277539.
14. Suwanbamrung C, Saengsuwan B, Sangmanee T, Thrikaew N, Srimoung P, Maneerattanasak S. Knowledge, attitudes, and practices towards dengue prevention among primary school children with and without experience of previous dengue infection in Southern Thailand. One Health. 2021;13:100275. doi:10.1016/j.onehlt.2021.100275
15. Vongpunsawad S, Intharasongkroh D, Thongmee T, Poovorawan Y. Seroprevalence of antibodies to dengue and chikungunya viruses in Thailand. PLoS One. 2017 Jun 29;12(6):e0180560.
16. Khongwichit S, Chansaenroj J, Thongmee T, Benjamanukul S, Wanlapakorn N, et al. (2021) Large-scale outbreak of Chikungunya virus infection in Thailand, 2018–2019. PLOS ONE 16(3): e0247314. https://doi.org/10.1371/journal.pone.0247314
17. Hayes RJ, Bennett S. Simple sample size calculation for cluster-randomized trials. Int. J. Epidemiol. 1999; 28: 319-326.
