# Supplementary material for: Seroprevalence and preventive practices of dengue and chikungunya among school children in Bangkok: Gaps in prevention and vaccination strategies
Source: PLoS Negl Trop Dis. 2026 Mar 16;20(3):e0013026. doi: 10.1371/journal.pntd.0013026 (PMC13004517; doi:10.1371/journal.pntd.0013026)
Supplement: S1 STROBE Checklist — The Strengthening the Reporting of Observational Studies in Epidemiology (STROBE)statement: guidelines for reporting observational studies. Lancet. 2007 Oct 20‌‌;370(9596):1453–7. PMID: 18064739. (DOCX) [file pntd.0013026.s002.docx]

# STROBE Checklist for Cross-Sectional Studies

| **Item No.** | **Recommendation** | **Page No. in Manuscript** | **Sentences/texts** |
| --- | --- | --- | --- |
| **Title and abstract** |  |  |  |
| Item no. 1 | 1. Indicate study’s design with a commonly used term in the title or abstract | Page 3 | A cross-sectional descriptive study |
|  | 1. Provide informative and balanced summary of what was done and found | Page 3 | - Children aged 10 to 15 years were included. Seroprevalence was determined using rapid diagnostic tests - Parents completed KAP questionnaires, including factors influencing vaccination decisions - From June to August 2024, 937 participants were enrolled   seroprevalence of dengue was 28.1% while that of chikungunya was 6.3% |
| **Introduction** |  |  |  |
| **Background/****rationale**  Item no. 2 | Explain the scientific background and rationale for the investigation being reported | Page 5-6 | - Dengue and chikungunya, both transmitted by *Aedes* mosquitoes, continue to pose significant public health concerns in Thailand. Despite ongoing vector control efforts, the incidence of infection remains high - A systematic review reported seroprevalence among Thai children aged 2-17 years, assessed by the plaque reduction neutralization test (PRNT), reaching up to 80% in certain regions - Chikungunya outbreaks have been more frequent in Thailand in southern regions. A study in 2017 reported a seropositivity rate of Chikungunya IgG antibodies by commercial enzyme-linked immunosorbent assays (ELISA) of 3% among individuals aged 10–19 in central Thailand. However, data on Chikungunya seroprevalence in Thai children remains limited. - Parental engagement in vector control efforts may further strengthen prevention efforts, and access to vaccination and other interventions also impacts disease control efforts |
| **Objectives**  Item no. 3 | State specific objectives, including prespecified hypotheses | Page 6 | - This study aims to determine the seroprevalence of dengue and chikungunya among children in Bangkok and assess parental KAP regarding disease prevention |
| **Method** |  |  |  |
| Study design  4 | Present key elements of study design early in the paper | Page 7 | A cross-sectional observational study. |
| Setting  5 | Describe the setting, locations, and relevant dates, including periods of recruitment, exposure, follow-up, and data collection | Page 7, 11 | - study was conducted in 12 schools in Bangkok. - Schools were selected from each region after initial contact and agreement to participate - The study was conducted in Bangkok during June-August 2024 |
| Participants  6 | (*a*) Give the eligibility criteria, and the sources and methods of selection of participants | Page 7 | - The study included children aged 10-15. - Children with medical conditions that could interfere with blood sampling on the study day were excluded. - The study was conducted on school premises, where parents provided consent for their children’s participation.   (all participant with parental consent were enrolled) |
| Variables  7 | Clearly define all outcomes, exposures, predictors, potential confounders, and effect modifiers. Give diagnostic criteria, if applicable | Page 10 | - The seroprevalence, defined by positive IgG - The history of dengue vaccination was reported as a percentage (Effect modifier) |
| Data sources/ measurement  8* | For each variable of interest, give sources of data and details of methods of assessment (measurement). Describe comparability of assessment methods if there is more than one group | Page 9 | - Rapid diagnostic tests were used to detect antibodies. The Abbott DENGUE IgG/IgM (Abbott Laboratories, USA) was used for dengue, while the Citest IgG/IgM - CITEST was used for chikungunya. - Both tests utilize   immunochromatography, requiring only 10 microlites for IgG and IgM detection.   - The reported sensitivity and specificity for the dengue test are 94.2% and 96.4%,   while for the chikungunya test, they are 90.3% and 99.9% for diagnosing acute  illness, respectively.   - Blood samples were collected using a capillary tube and   transferred to the test kit with buffer, and results were available in 15-20 minutes. |
| Bias  9 | Describe any efforts to address potential sources of bias | Page 23 | (also in discussion part) |
| Study size  10 | Explain how the study size was arrived at | Page 10 | Sample size calculation based on prevalence data |
| Quantitative variables  11 | Explain how quantitative variables were handled in the analyses. If applicable, describe which groupings were chosen and why | Page 10 | - Seroprevalence was calculated overall and by region with 95% confidence intervals (CI). - Vaccine knowledge and willingness to vaccinate were presented as percentages. |
| Statistical methods  12 | 1. Describe all statistical methods, including those used to control for confounding 2. Describe any methods used to examine subgroups and interactions 3. Explain how missing data were addressed 4. If applicable, describe analytical methods taking account of sampling strategy 5. Describe any sensitivity analyses | Page 10 | - Differences between age groups and regions were analyzed using Chi-square tests and one-way ANOVA. |
| Result |  |  |  |
| Participants  13* | 1. Report numbers of individuals at each stage of study—eg numbers potentially eligible, examined for eligibility, confirmed eligible, included in the study, completing follow-up, and analysed 2. Give reasons for non-participation at each stage 3. Consider use of a flow diagram | Page 11-15 | - a total of 937 students were enrolled (for seroprevalence) - The questionnaire study included responses from 889 participating parent |
| Descriptive data  Outcome data  14* | 1. Give characteristics of study participants (eg demographic, clinical, social) and information on exposures and potential confounders 2. Indicate number of participants with missing data for each variable of interest | Table 1  Page 12-13 | - mean age of seroprevalence stuy is 11 years (SD: 1.6) - The questionnaire study included responses from 889 participating parents, representing a 95% response rate. The mean age of respondents was 42 years (SD: 8.8). - 65%) had resided in their current neighborhood for over five years - 34% of participants lived in detached houses, 25% in condominiums or apartments, 19% in townhouses, |
| Outcome  15 | Report numbers of outcome events or summary measures | Page 13, 15  Table 1, Figure 2, 3 | Seroprevalence of dengue and chikungunya   - **Total participants:** 937 children aged 10–15 years - **Dengue IgG positive:** 264 participants (28**% seroprevalence**) - **Chikungunya IgG positive:** 59 participants (6**% seroprevalence**)   KAP study   - high level of awareness regarding dengue and chikungunya transmission and symptoms, with 71-94% correctly responding to dengue-related questions - 69-81% correctly answering chikungunya-related items - The primary factors influencing vaccine uptake were safety (65-68%) and efficacy (58-61%) |
| Main results  16 |  | Pages 13-18  (Table 2, Figure 2, 3) | Serology   - The overall seroprevalence, determined by positive IgG results, was 28.1% (95% CI: 25.2–31.0) for dengue and 6.3% (95% CI: 4.7–7.9) for chikungunya - KAP scoring and factors influence vaccination as in figure 2,3 |
|  | 1. Give unadjusted estimates and, if applicable, confounder-adjusted estimates and their precision (eg, 95% confidence interval). Make clear which confounders were adjusted for and why they were included 2. Report category boundaries when continuous variables were categorized 3. If relevant, consider translating estimates of relative risk into absolute risk for a meaningful time period | Page 13  Table 2, Figure 2,3 |  |
| Other analyses  17 | Report other analyses done—eg analyses of subgroups and interactions, and sensitivity analyses | Page 11-13  (Table 1 and Figure 2) | - Regional variations in seroprevalence were observed, though these differences were not statistically significant. - Dengue seroprevalence exhibited a tendency to increase with age, whereas no such trend was observed for Chikungunya seroprevalence |
| **Discussion** |  |  |  |
| Key result  18 | Summarise key results with reference to study objectives | Page 20-21 | - The observed dengue seroprevalence of 28.1% and chikungunya seroprevalence of 6.3%, as determined by rapid diagnostic tests, underscore the continued transmission of these arbovial infections in Bangkok - The KAP assessment demonstrated a high level of awareness regarding dengue and chikungunya among parents, with most recognizing these diseases as significant public health concerns. - Despite high awareness, gaps in preventive practices were evident |
| Limitations  19 | Discuss limitations of the study, taking into account sources of potential bias or imprecision. Discuss both direction and magnitude of any potential bias | Page 23 | - The study population was not evenly distributed across Bangkok, which may limit the generalizability of the findings to the entire city (selection bias) - the use of rapid diagnostic tests, which have lower sensitivity compared to ELISA or PRNT, may have underestimated seroprevalence (measurement bias) |
| 20  Interpretation | Give a cautious overall interpretation of results considering objectives, limitations, multiplicity of analyses, results from similar studies, and other relevant evidence | Page 23  (conclusion) | - Dengue and chikungunya infections remain significant public health concerns in Thailand, with annual outbreaks and a rising chikungunya trend. Despite high awareness of these diseases, preventive practices are not consistently adopted, likely due to urban living constraints and socio-economic factors. The low uptake of dengue vaccination, despite its demonstrated efficacy, underscores the need for stronger public health initiatives to promote vaccine acceptance and accessibility. |
| 21  Generalisability | Discuss the generalisability (external validity) of the study results | Page 24 | - However, given the rising chikungunya burden in Thailand, post-outbreak seroprevalence studies and consideration of target vaccination strategies are warranted. - This finding underscores the need for additional strategies, such as vaccination, to reduce the disease burden effectively |
| Other information |  |  |  |
| 22  Funding | Give the source of funding and the role of the funders for the present study and, if applicable, for the original study on which the present article is based | - | This research was funded by the Ratchadapiseksomphot Fund, Faculty of Medicine, Chulalongkorn University and Pasteur Institute, France. |
