# Supplementary material for: Seroprevalence and preventive practices of dengue and chikungunya among school children in Bangkok: Gaps in prevention and vaccination strategies
Source: PLoS Negl Trop Dis. 2026 Mar 16;20(3):e0013026. doi: 10.1371/journal.pntd.0013026 (PMC13004517; doi:10.1371/journal.pntd.0013026)
Supplement: Attachment — Submitted filename: Response to Reviewers 23Aug2025.docx [file pntd.0013026.s003.docx]

11 August 2025

Dear Editor and Reviewers,

Revised of the submitted manuscript entitled “Seroprevalence and preventive practices of dengue and chikungunya among school children in Bangkok: Gaps in prevention and vaccination strategies” (PNTD-D-25-00495).

We would like to express our sincere gratitude for the time and effort you have dedicated to reviewing our manuscript. We appreciate the valuable feedback provided, which has helped us to improve the clarity and rigor of our work.

We have carefully considered all comments and suggestions and have made the following revisions:

Reviewers' Comments:

Methods
Reviewer #1:

1. The objectives of the study are clearly articulated, aiming to assess the seroprevalence of dengue and chikungunya infections among children aged 10-15 years in Bangkok and evaluate the knowledge, attitudes, and practices (KAP) related to mosquito-borne disease prevention. However, the hypothesis could be more explicitly stated.

Response to reviewers:

Thank you for your valuable comment. We appreciate your suggestion and agree that clearly stating the hypothesis would strengthen the clarity of the study framework. We have now revised the manuscript to state the following hypotheses explicitly:

- A substantial proportion of children aged 10–15 years in Bangkok have been previously exposed to dengue and/or chikungunya virus, as indicated by the presence of specific IgG antibodies.
- There is an association between children’s knowledge, attitudes, and practices (KAP) regarding mosquito-borne disease prevention and their serostatus for dengue and chikungunya.

Revision added: **Introduction part, page 6-7, line 114-124**

This study aims to determine the seroprevalence of dengue and chikungunya virus infections among children aged 10–15 years in Bangkok and to assess their parents' KAP related to mosquito-borne disease prevention. Understanding both the immunological exposure and behavioral factors in this age group is critical for informing targeted public health strategies. The study is based on the following hypotheses: (1) a substantial proportion of children have serological evidence of prior dengue and/or chikungunya virus infection, and (2) higher levels of knowledge, positive attitudes, and appropriate preventive practices are associated with a lower likelihood of seropositivity. These objectives and hypotheses are intended to guide both data interpretation and the development of future school- and community-based interventions.

2. The cross-sectional descriptive study design is appropriate for addressing the stated objectives. However, the reliance on rapid diagnostic tests (Abbott DENGUE IgG/IgM and Citest Chikungunya IgG/IgM) raises concerns about the accuracy of the seroprevalence data.

The statistical analyses used to support the conclusions are appropriate. However, the limitations of the diagnostic tests used should be considered in the interpretation of the results.

Response to reviewers:

Thank you for your comment. We agree on the limitations of using rapid diagnostic tests (RDTs) in estimating seroprevalence. As noted in the revised Discussion, RDTs generally have lower sensitivity and specificity compared to laboratory-based assays such as ELISA or PRNT. For example, the Abbott Bioline Dengue IgG/IgM test has been reported to have a sensitivity of approximately 21% when detecting past dengue infections compared with ELISA. Despite these limitations, our choice of RDTs was driven by feasibility considerations in a school-based field setting, as RDTs allow for minimally invasive fingertip blood collection and do not require venipuncture, trained phlebotomists, or laboratory infrastructure. Moreover, in light of the recommendations to use vaccines only in individuals with previous natural exposure to DENV, the use of RDTs will likely be the preferred choice of test as compared to the more laborious use of ELISA. We have expanded the discussion to state these limitations clearly, reference comparative sensitivity data, and emphasize that our findings should be interpreted as conservative estimates.

Revision added:

**Discussion part, page 21, line 335-345**

The dengue seroprevalence observed in this study is comparable with seroprevalence by the RDTs but lower than by the ELISA method reported in other parts of Thailand. For example, a 2021 study in Ratchaburi province, an urban center in Western Thailand, found a seroprevalence of 24.3% in the 9-14 year age group using the SD Bioline Dengue Duo NS1 Ag & IgG/IgM RDT, and 60.5% using ELISA^.(17)^ Similarly, another study in 2023 in 4 urban centers across Thailand (Nonthaburi, Phisanulok, Udon Thani, and Surat Thani Province) estimated a seroprevalence of 55.4% (95%CI 54.3-56.4) in the 10-14 age group using ELISA.^(16)^ Similar seroprevalence rates were observed using ELISA on samples from children (10-15 years of age) in urban and rural sites in North Eastern Thailand. ^(18)^

**Discussion part, page 26, line 423-446**

Additionally, the use of rapid diagnostic tests (RDTs) for detecting dengue and chikungunya antibodies may have led to an underestimation of true seroprevalence, particularly for past infections where antibody titers decline over time. RDTs typically have lower sensitivity compared to enzyme-linked immunosorbent assays (ELISA) or plaque reduction neutralization tests (PRNT). For example, the Abbott Bioline Dengue IgG/IgM test has been reported to demonstrate a sensitivity of only 21% when compared to ELISA in detecting past dengue infections^(23)^. Another study in a similar age group in Ratchaburi province compared RDT-based dengue seroprevalence with ELISA-based data and reported that the ELISA estimate was approximately 2.5 times higher than the RDT estimate, underscoring the substantial difference in detection rates between these methods. ^(15)^ ELISA-based studies from 2020 and 2023 reported seroprevalence rates of 59.7%⁽^17^⁾ and 55.4%⁽^16^⁾, respectively, in similar age groups. Similarly, the Citest Chikungunya IgG/IgM test is designed primarily for use in the acute phase of infection and may exhibit cross-reactivity with antibodies against other flaviviruses. Despite these limitations, the selection of RDTs was based on practical considerations related to field implementation in a school-based setting. RDTs require only a small volume of fingertip blood, are easy to use in non-laboratory environments, and avoid the need for venipuncture, which can be logistically and ethically challenging in school-aged populations. Moreover, in light of the recommendations to use vaccines only in individuals with previous natural exposure to DENV, the use of RDTs will likely be the preferred choice of test as compared to the more laborious use of ELISA. Future studies using more sensitive and specific laboratory assays such as ELISA or PRNT are, however, warranted to obtain more accurate estimates of seroprevalence and confirm these preliminary findings.

3. The population is clearly described, including children aged 10-15 years from 12 schools in Bangkok. The sample size of 937 participants appears sufficient to ensure adequate power for dengue but is close to the required size for chikungunya.

Response to reviewers: Thank you for highlighting this point. We based our calculation on previously reported dengue seroprevalence of 55%, which produced the largest required sample size among the two viruses studied. Although chikungunya seroprevalence is much lower (around 3%), a low prevalence substantially reduces statistical power, even if the absolute sample size needed for simple prevalence estimation is smaller. For instance, with 380 participants, the study would have adequate power for dengue (~82%) but very low power for chikungunya (~20%), limiting the reliability of prevalence estimates for the latter. In other words, while dengue seroprevalence determined the minimum required sample size, chikungunya estimates benefited from the larger enrollment by achieving much higher precision and statistical power. Because this investigation was conducted as a substudy of a larger “geomosquito” project, we were able to enroll 937 participants—well above the minimum needed. This larger sample size maintained excellent statistical power for dengue and increased power for chikungunya to >99%, enabling more robust and precise estimates for both viruses.

Revision added: **Method, Sample size calculation, page 10-11, line 178-185**

The sample size was determined based on expected seroprevalence rates of dengue and chikungunya from previous studies. To ensure adequate statistical power for both dengue and chikungunya, 380 participants were sufficient for dengue (assuming 55% seroprevalence for dengue and 3% for chikungunya). However, this study was a substudy of a larger study, which included at least 840 participants; we enrolled according to the main study. This final sample size provided excellent power for dengue and still delivered high statistical power for chikungunya.

Reviewer #2:

4. Sample size calculation: Details provided here are good for a proposal but not necessarily so for a scientific manuscript. You may need to summarize it.

Response to reviewers: Thank you for pointing this. We revised this part.

Revision added: Same as Comment 3 of reviewer#1

Reviewer #3:

5. The methodology used for sampling have a serious error on the distribution pattern of the samples selected. The study was carried out in 5 zones of Thailand viz., Northern, Southern, Eastern, Western, and Central. However, authors chose 12 schools agreed to participate from these sites one, six, one, two, six and two respectively. As a result, the samples collected from these regions were also show wide variations in seroprevalence data for Dengue. Hence, the results obtained in the study may have some range of ambiguity, when generalized for the Country. (Authors state these as limitations in the study).

Response to reviewers: Thank you for your insightful comment. This study was not conducted across all of Thailand but in six administrative zones of Bangkok, the capital city of Thailand, which cover approximately 1,569 km^2^. The zones were defined by the Bangkok Metropolitan Administration GIS portal (https://district.bangkok.go.th/SEDPortal/50-map-overview/#tab-41f3183fa0f962177eb). We acknowledge that the sampling distribution across zones in Bangkok was uneven, largely due to the voluntary participation of schools and operational feasibility constraints. As a result, certain zones were overrepresented while others had fewer participants, which may have introduced bias and contributed to variability in seroprevalence estimates. This limitation has been explicitly noted in the Discussion, and we have emphasized that the findings should be interpreted with caution, particularly when extrapolating beyond Bangkok. Future studies using stratified random sampling across zones are warranted to provide more representative estimates.

Revision added: **Discussion, limitation part, Page 25-26, line 414-423**
The sampling distribution across Bangkok’s geographic zones was not uniform because school recruitment was based on voluntary participation and operational feasibility. Consequently, some zones were overrepresented while others contributed fewer samples, potentially introducing bias and contributing to observed variability in seroprevalence across zones. This uneven representation limits the generalizability of the findings to the broader population of Bangkok or to other regions in Thailand. However, the study does provide preliminary insights into dengue and chikungunya exposure among school-aged children across Bangkok. These findings should be interpreted as exploratory data to inform future, more representative surveillance and intervention planning.

--------------------------------------------------------------------------------------------------------------------------------------------

Results
Reviewer #1:

6. The analysis presented matches the analysis plan. However, the results may be compromised by the limitations of the rapid diagnostic tests used.

Response to reviewers: Thank you for your comment. As noted in our previous response (Comment 2), we fully acknowledge the limitations of the rapid diagnostic tests used in this study, particularly their lower sensitivity compared to ELISA or PRNT methods. These limitations have been clearly documented in the **Discussion** section under the limitations paragraph. We also explained the rationale for selecting rapid tests based on feasibility for school-based implementation, and emphasized the need for future studies using more sensitive assays.

Reviewer #2:

7. Table 1: The kit picks IgG/IgM, so why is only IgG being reported here?

Response to reviewers: Thank you for your comment. While the rapid diagnostic test detects both IgG and IgM, our analysis focused on IgG results only. This decision was based on two main reasons:
(1) The study population consisted of healthy, asymptomatic school children, where isolated IgM positivity is more likely to represent cross-reactivity or false positives rather than true acute infection.
(2) Our study aimed to assess seroprevalence as an indicator of past exposure; therefore, only IgG results were considered in accordance with our study protocol.

We have clarified this rationale in the manuscript to ensure transparency.

Revision added: **Method part; Page 11, line 189-193**

The seroprevalence, defined as a positive IgG result, was calculated overall and stratified by region, with 95% confidence intervals (CI). As the study included only healthy, asymptomatic individuals, isolated IgM-positive results were not included in the analysis due to the potential for cross-reactivity and limited specificity for past infection.

8. Prevalence of dengue and chikungunya infections (line 218-219): As this was a cross-sectional study, trends over time cannot be assessed. The statement should be revised to reflect a single point-in-time observation rather than implying a trend.

Response to reviewers: Thank you for your helpful comment. We agree that, given the cross-sectional design, temporal trends cannot be inferred. We have revised the sentence to reflect differences in seroprevalence **between age groups** rather than suggesting a trend over time.

Revision added: **Result part, page 14, line 241-243**

Dengue seroprevalence was slightly higher among older age groups, whereas chikungunya seroprevalence remained relatively consistent across age groups.

9. Knowledge, attitude and practice regarding dengue and chikungunya (line 245): How many doses of the vaccine did they receive?

Response to reviewers: Of the 56 participants, 32 (57%) received one dose, 19 (34%) received two doses, and 4 (7%) received three doses of the dengue vaccine; one (2%) participant did not report the number of doses received.

Revision added: **Result part, page 16, line 268-271**

Of the 56 participants, 32 (57%) received one dose, 19 (34%) received two doses, and 4 (7%) received three doses of the dengue vaccine; one (2%) participant did not report the number of doses received.

10. Line 272-274: Seroprevalence was measured in school children, while information on preventive measures was collected from parents. How were the two datasets linked to support the inferences made? For example, you mentioned that some parents use mosquito repellents before going out, but it was not specified whether these repellents were also used on their children. These assumptions need to be clarified to strengthen the validity of this statement.

Response to reviewers: Thank you for this important point. The serological data were collected from the children, while the information on preventive measures was reported by their parents or guardians. We linked the datasets at the individual level, matching each child’s serostatus with their own parent’s reported preventive practices. However, we acknowledge that parental reports may not fully reflect the actual preventive behaviors applied to the children themselves (e.g., use of repellents on children). To address this, we have clarified in the manuscript that the analysis reflects the association between parental-reported household preventive measures and children’s seroprevalence, and we acknowledge the limitation that direct child-specific preventive practices were not separately measured.

Revision added:

**Result part, page 18, line 296-299**

Seroprevalence data from children were analyzed in relation to preventive measures reported by their parents or guardians. There was no statistically significant difference in seroprevalence between children whose parents reported regularly implementing household preventive measures and those who did not.

**Discussion/limitation part, page 27, line 446-452**

It is important to note that preventive measures were reported by parents or guardians at the household level and may not fully represent the actual preventive behaviors specifically applied to the children, such as the use of mosquito repellents directly on them. This limitation may affect the accuracy of associations between preventive practices and seroprevalence, and should be considered when interpreting these findings. Future studies could benefit from directly assessing child-specific preventive behaviors.

Reviewer #3: Results are presented well

--------------------------------------------------------------------------------------------------------------------------------------------

Discussion and Conclusions
Reviewer #1:

11. The conclusions are not supported by the data presented. There are no limitations of the diagnostic tests described and the significant concerns regarding their accuracy for seroprevalence studies should be more thoroughly addressed.
    The authors discuss how the data can advance understanding of dengue and chikungunya seroprevalence and preventive practices. However, the potential inaccuracies in the seroprevalence data may limit the study's contributions.
    The public health relevance is addressed, emphasizing the need for enhanced community education and vector control interventions.

Response to reviewers: Thank you for your valuable feedback. We acknowledge that the limitations of the rapid diagnostic tests used in this study may affect the accuracy of seroprevalence estimates. We have now more explicitly addressed these limitations in the Discussion and Conclusion sections, including the potential underestimation of seroprevalence compared to standard laboratory assays such as ELISA or PRNT. We agree that these constraints may limit the generalizability of the seroprevalence data and, by extension, the study’s broader public health implications. Nonetheless, we believe that the study provides important preliminary insights into dengue and chikungunya exposure and preventive practices in a school-aged population, highlighting areas for targeted education, vector control, and vaccine promotion. We have revised the Conclusion to better reflect these points.

Revision added: **Conclusion, Page 27, line 456-469**

Dengue and chikungunya infections remain important public health concerns in Thailand. Our findings suggest that age-related exposure to dengue and early signs of chikungunya transmission are prevalent among school-aged children in Bangkok. While rapid diagnostic tests are limited in sensitivity, particularly for detecting past infections, and may underestimate the true seroprevalence, their ease of use in a school setting provides valuable field-level insights into exposure trends and community preventive practices. These data should be interpreted as exploratory, highlighting the need for more rigorous serological assessments using ELISA or PRNT. The low uptake of dengue vaccination, despite awareness, highlights the need for strengthened public health messaging and improved vaccine accessibility. These findings underscore the importance of integrated community education and vector control strategies to mitigate the burden of mosquito-borne diseases in Thailand.

Reviewer #2:

12. Line 302: ‘arbovirus’, correct the spelling

Response to reviewers:

Thank you for your comment. We edit this word.

13. Line 312-315: Misleading interpretation:
    • If only 46% of those self-reporting past dengue tested positive, it doesn't mean seroprevalence is higher—it may actually suggest asymptomatic infections or recall bias.
    • Conversely, if you're suggesting that many who didn’t report previous dengue still had IgG antibodies, that could suggest underreporting and higher seroprevalence—but this isn't what the sentence says. Rephrase for clarity and correctness.

Response to reviewers:

Thank you for your insightful comment. We agree that the previous phrasing may have led to confusion. Our intention was to highlight the discrepancy between self-reported dengue history and the observed seropositivity. We now acknowledge that this could reflect either underreporting due to recall bias or a high rate of asymptomatic infections. We have revised the sentence to clarify this point and improve accuracy.

Revision added: **page 22, line 350-353**

Among those who self-reported previous dengue infection, only 46% tested positive for dengue IgG. This discrepancy may reflect waning antibody levels not detectable by the rapid test, recall bias, or a significant proportion of asymptomatic or unrecognized infections in the broader population.

14. Line 322: ‘research’, it has no plural form

Response to reviewers: We edit the word per your suggestion.

15. Line 338: The questionnaire assessed the ‘use’ of repellents, not ‘access’ to them. Please revise the wording accordingly to accurately reflect what was measured.

Response to reviewers: Thank you for pointing this out. We agree that the original wording was inaccurate. The questionnaire assessed actual use rather than access to mosquito repellent. We have revised the sentence to reflect this and to better align with the data collected.

Revision added: Discussion part, page 23, line 456-460

Despite high awareness, gaps in preventive practices were evident. Barriers such as limited use of mosquito repellent, lack of community-wide control initiatives, and socioeconomic constraints may hinder the consistent adoption of preventive behaviors.

16. Line 347: Clarify if the children took one or two doses of the vaccine.

Response to reviewers: Among these, 32 (57%) received one dose, 19 (34%) received two doses, and 4 (7%) received all three doses; one participant (2%) did not report the number of doses. Some participants received the earlier dengue vaccine, which follows a three-dose schedule.

Revision added: **Discussion part, page 24, line 386-392**

Despite relatively high awareness of dengue vaccines among the study population, vaccine uptake remained low, with only 7% of children having received at least one dose. Among those vaccinated, most had not completed the full vaccination schedule: 58% received only one dose, 34% received two doses, and 7% received three doses, while one participant did not report the number of doses received. This finding may reflect limited access, vaccine hesitancy, or lack of awareness regarding the recommended three-dose schedule for some dengue vaccines.

17. Line 381-382: Data provided does not support this assumption; hence, it is advisable to leave it out of your conclusion.

Response to reviewers: Thank you for your comment. We agree with your observation. The sentence in question has been removed from the conclusion to ensure that our interpretations remain strictly evidence-based and supported by the data presented.

Reviewer #3:

18. As mentioned above as the study does not follow a statistically sound procedure on the distribution of samples, the results obtained may not be generalized for the Country. Besides, the specificity and sensitivity of the IgG kits used to estimate the seroprevalence of Dengue and Chikungunya may have to be justified by previous studies carried out using these kits if any. Authors have provided only references of the firms that manufactured the kits.

Response to reviewers:

We appreciate your valuable feedback. We acknowledge that the sample distribution was based on voluntary school participation, but the study was conducted in Bangkok and not across the whole of Thailand. We have given more information of other studies both across Thailand and within Bangkok. The limitation of intra-urban sampling has been clearly stated in the manuscript.

Regarding the diagnostic tests, we have also addressed their limitations in the discussion section. Specifically, we noted that the sensitivity of the Abbott Bioline Dengue IgG/IgM rapid diagnostic test is reported to be approximately 21% when compared with ELISA, and that the chikungunya rapid test is designed primarily for use during the acute phase and may exhibit cross-reactivity with other flaviviruses. We have cited relevant literature to support this and revised the manuscript accordingly. We also revised the conclusion in the documented limitations of interpretation of our study.

Revision added: **Conclusion, page 27-28, line 457-469**

Dengue and chikungunya infections remain important public health concerns in Thailand, with dengue seroprevalence increasing with age and evidence of past chikungunya exposure. While the use of rapid diagnostic tests and may underestimate the true seroprevalence, these findings still offer useful insights into regional exposure patterns and community preventive practices. The low uptake of dengue vaccination, despite awareness, highlights the need for strengthened public health messaging and improved vaccine accessibility. These findings underscore the importance of integrated community education and vector control strategies to mitigate the burden of mosquito-borne diseases in Thailand.

--------------------------------------------------------------------------------------------------------------------------------------------

Editorial and Data Presentation Modifications
Reviewer #1: (No Response)

Reviewer #2: None

Reviewer #3:

19. Discussion: Line 321 Aedes aegypti may be corrected as Aedes albopictus, as E1 A226V mutation provides transmission advantage to the latter species

Response to reviewers: We edited as your suggestion.

Revision added: **Discussion, page 22, line 357-359**

This outbreak, particularly prominent in Bangkok, was associated with mutations in chikungunya virus strains, such as the E1-A226V mutation, which enhances viral adaptation to *Aedes albopictus* mosquitoes.

--------------------------------------------------------------------------------------------------------------------------------------------

Summary and General Comments
Reviewer #1: I have carefully reviewed the article titled "Seroprevalence and preventive practices of dengue and chikungunya among school children in Bangkok: Gaps in prevention and vaccination strategies" submitted for publication in PLOS Neglected Tropical Diseases.
The study aims to assess the seroprevalence of dengue and chikungunya infections among children aged 10-15 years in Bangkok and evaluate the knowledge, attitudes, and practices (KAP) related to mosquito-borne disease prevention. The methodology involves a cross-sectional descriptive study conducted across 12 schools in Bangkok, using rapid diagnostic tests (Abbott DENGUE IgG/IgM and Citest Chikungunya IgG/IgM) based on the immunochromatography technique.
This article presents potentially valuable information because it aims to address a significant public health concern in Thailand, focusing on the seroprevalence of Chikungunya and dengue in vaccine eligible school children. The inclusion of KAP assessments provides valuable insights into community awareness and preventive practices. In addition, the study's sample size (937 participants) appears to be adequate to support the findings for Dengue and very close to those required for Chikungunya.
However, I have the following major concerns with this manuscript:

20. The primary concern with this study is the reliance on rapid diagnostic tests (Abbott DENGUE IgG/IgM and Citest Chikungunya IgG/IgM) to determine seroprevalence. These tests are designed for the qualitative detection of anti-DENV IgM and IgG antibodies to aid in distinguishing acute primary versus secondary infections. However, seroprevalence studies require accurate, quantitative (or at least highly sensitive) measurement of past exposure across all age groups. Several key limitations prevent the Bioline RDT from reliably estimating true IgG seroprevalence:
    • The Abbott Bioline Dengue IgG/IgM has low field sensitivity for detecting past (IgG) infections: Studies such as Arkell P, et. al. (2022). Field evaluation of rapid diagnostic tests to determine dengue serostatus in Timor-Leste. PLoS Negl Trop Dis 16(11): e0010877, have shown that the Bioline IgG/IgM test has low sensitivity (21.1%) for detecting past IgG infections compared to reference IgG ELISA tests.
    • The Abbott Bioline Dengue IgG/IgM and the Citest Chikungunya IgG/IgM cassette were optimized for the detection of acute-phase antibodies: RDTs are optimized to detect high-titer IgM and acute/secondary IgG boosts, rather than low, stable IgG levels months to years after infection. This can result in false-negative results for individuals with remote infections. The use of rapid diagnostic tests, which have lower sensitivity compared to ELISA or PRNT, may have underestimated seroprevalence, particularly for past infections where antibody levels decline over time. No data was presented in this manuscript demonstrating that the test was validated for determining seroprevalence of past infections with more sensitive methods.
    • Potential Cross-Reactivity: Cross-reactivity with other flaviviruses can lead to misclassification and should be addressed.

    While the study provides valuable insights into the seroprevalence of dengue and Chikungunya among school children in Bangkok, the use of rapid diagnostic tests with known limitations for seroprevalence studies raises concerns about the accuracy of the findings. For reliable estimation of age-specific or population-level dengue seroprevalence, laboratory-based IgG ELISAs or Plaque Reduction Neutralization Tests (PRNT) remain the gold standards. Therefore, I recommend reconsidering the publication of this study until more accurate diagnostic methods are employed to validate the seroprevalence data.

Response to reviewers: We agree with the reviewer that RDTs, such as those used in our study, are not gold-standard for determining true seroprevalence and may underestimate past exposure due to lower sensitivity, particularly for remote infections. As mentioned in our revised discussion and conclusion, our study aims to provide an exploratory, field-based perspective on exposure and prevention behaviors among school children. We have clarified these limitations more explicitly and revised the title and conclusion to reflect the preliminary nature of our findings. We hope this transparency and framing clarify the intent and appropriate interpretation of our results.

Reviewer #2:

21. This manuscript offers valuable and novel insights into the topical issue of dengue and chikungunya infections among school children in Bangkok. It also explores parental knowledge, attitudes, and practices, uncovering findings with potential implications for policy and improved disease control strategies in the region. However, the manuscript would benefit from thorough English language editing to address grammatical and stylistic issues. Overall, this work makes a meaningful contribution to the body of knowledge in this field.

Response to reviewers: We appreciate the comment and have revised and edited English language.

Reviewer #3:

22. Sampling errors and methodological ambiguity exist in the study. Hence the results may not represent the true picture of seroprevalence of Dengue and Chikungunya in Thailand.

Response to reviewers:

We appreciate the reviewer’s observation. We fully acknowledge that our sampling approach—based on voluntary participation from schools—resulted in an uneven distribution of participants across geographic zones. This limitation, which we have clearly stated in the Discussion section (Page 25, Lines 479–487), may affect the representativeness and generalizability of our findings at the national level. Our intention was not to provide a nationally representative seroprevalence estimate, but rather to offer preliminary, school-based insights into regional exposure patterns and preventive behaviors among children in Bangkok and surrounding areas.

Despite this limitation, we believe the study provides valuable field-level data and highlights important gaps in prevention and vaccination practices. We have now revised the manuscript title and conclusion to better reflect the scope and exploratory nature of our work. Future research employing systematic or stratified random sampling across regions and more sensitive diagnostic assays (e.g., ELISA, PRNT) is warranted to validate and expand upon these findings.

Sincerely Yours,

Watsamon Jantarabenjakul
